# High prevalence and multilevel correlates of suicidal ideation Among Bahamian adolescents: findings from a national study

**DOI:** 10.3389/frcha.2026.1848813

**Published:** 2026-07-16

**Authors:** Deogwoon Kim, Jessica Moszkowicz, Nikkiah Forbes, Marcellus Taylor, Chloee Deveaux, Karen MacDonell, Lynette Deveaux, Bo Wang

**Affiliations:** 1 Department of Population and Quantitative Health Sciences, UMass Chan Medical School, Worcester, MA, United States; 2 UMass Chan Medical School, Worcester, MA, United States; 3 National HIV/AIDS Programme, Ministry of Health & Wellness, Nassau, Bahamas; 4 Government and Public Policy Institute, University of The Bahamas, Nassau, Bahamas; 5 English Public Texts Department, Trent University, Peterborough, Ontario, Canada; 6 Department of Behavioral Sciences and Social Medicine and Center for Translational Behavioral Science, Florida State University, Tallahassee, FL, United States

**Keywords:** cyberbullying, depression, digital risk factors, multilevel factor, suicidal ideation, caribbean, adolescents

## Abstract

**Introduction:**

Suicide is among the leading causes of death among adolescents in the Caribbean, yet suicidal ideation among Bahamian adolescents remains understudied. Few studies have assessed the association between online risk behaviors, environmental factors, and suicidal ideation. This study investigated the prevalence of suicidal ideation among Bahamian adolescents and examined its multilevel correlates, including offline and online risk behaviors and environmental factors.

**Methods:**

Grade 9 students attending public schools participating in a nationwide implementation trial in The Bahamas completed a survey during 2023-2024. A total of 2,357 students participated. Data were analyzed using generalized linear mixed-effects models.

**Results:**

Approximately 46% of students reported lifetime suicidal ideation, and among those, 33% reported self-harm thoughts nearly every day in the past two weeks. Female gender, engagement in cyberbullying, sexting and certain offline risk behaviors, and depressive symptoms were associated with increased suicidal ideation, whereas parental monitoring was protective. Neighborhood risk was not associated with suicidal ideation.

**Conclusion:**

These findings underscore the urgent need for multilevel suicide prevention strategies that address online and offline risk behaviors and strengthen targeted mental health support for adolescents in The Bahamas.

## Introduction

Suicide is the third leading cause of death among adolescents across the Region of the Americans with age-standardized suicide rates (per 100,000) ranging from 1.7 to 15.5 for boys and 1.6 to 5.1 for girls between 2000 and 2014 (1, 2). Suicidal ideation, a strong predictor of suicide attempts, is prevalent among adolescents in the Caribbean and Latin America (3, 4). However, data on adolescent suicidal ideation in The Bahamas are scarce. A study in 2013 reported that 12.5% of boys and 22.7% of girls in The Bahamas experienced suicidal ideation (4). Despite this concerning trend, suicide research and evidence-based prevention interventions in The Bahamas remain limited (5).

Understanding multilevel factors of adolescent suicidal ideation is critical for developing an effective prevention intervention. At the individual level, gender and psychopathological symptoms are well-established predictors of suicidal ideation. Systematic reviews have consistently found that adolescent girls report higher rates of self-harm thoughts and suicidal ideation than boys (6, 7). Adolescent stress often presents as internalizing symptoms (e.g., depressive mood) or externalizing behaviors (e.g., substance use, aggression, and sexual risk behavior), all of which increase the risk of suicidality (8). Furthermore, adolescents are increasingly exposed to online risks, such as cyberbullying and online sexual harassment, which have been associated with other risk behaviors and suicidal ideation (9, 10).

Beyond individual level, adolescents’ suicidal ideation can be either exacerbated or mitigated by their parents and surrounding environments. Parental monitoring can reduce self-harm thoughts and suicidal ideation (11), whereas perceived neighborhood risk is associated with increased suicidal ideation (12). The impact of residence, however, remains mixed. Some studies suggest that rural adolescents experience poorer mental health because of limited access to mental health resources (13), whereas others report higher suicidal ideation among urban adolescents (14), potentially because of greater environmental stress in urban settings (15).

Although several studies from the Caribbean have identified several correlates of adolescent suicidal ideation including gender, depression, offline risk behavior engagement, and parental involvement, available data remain limited in both geographic coverage and conceptual scope (16–18). Most Caribbean studies have focused primarily on individual-level factors, while environmental factors and the emerging issue of online risks have received little attention. Furthermore, country-specific evidence from The Bahamas is scare, limiting understanding of the multilevel factors that may facilitate or reduce adolescent suicidal ideation among Bahamian adolescents. Therefore, this study aimed to identify multilevel factors associated with suicidal ideation among Bahamian adolescents, including offline risk behaviors and online risk exposure as well as parental and environmental factors. The analysis was guided by the socioecological model, which emphasizes the complex interplay between individuals and contextual factors in shaping health behaviors (19). A better understanding of how these factors affect suicidal ideation can inform the development of comprehensive prevention strategies.

## Methods

### Data

Data were collected as part of a nationwide implementation trial of an evidence-based HIV prevention program in public schools in The Bahamas. All public junior high schools in The Bahamas participated in the nationwide implementation trial, and students in these schools completed the survey as part of the annual program assessment. Although the parent study collected longitudinal data, this study used a cross-sectional analysis collected from Grade 9 students. We focused on Grade 9 students as risk behaviors and mental health issues tend to peak during this late adolescent period (20). Grade 9 students completed the Bahamian Youth Health Risk Behavioral Inventory, adapted from the Youth Health Risk Behavioral Inventory and validated in The Bahamas (21). Students completed the survey anonymously in classrooms using a paper-and-pencil format during 2023-2024, under the supervision of research staff. At the beginning of the survey, students were informed that they could skip any questions or stop at any time if they felt uncomfortable. The survey included a contact number for discussing suicidal thoughts, and students with suicidal ideation were encouraged to reach out. School guidance counselors were available at schools to support students with high risk and refer students with acute distress to school psychologists for further evaluation and support. Institutional Review Board (IRB) approvals were obtained from UMass Chan Medical School and The Bahamas’ Princess Margaret Hospital, Public Hospitals Authority.

### Measures

*Lifetime suicidal ideation* was assessed with the question, “Have you ever seriously thought about killing yourself?” (yes/no). Guided by the socioecological model and exiting literature, independent variables were selected and organized across individual, interpersonal, and environmental levels. At the individual level, variables included internalizing symptoms, externalizing behaviors, and exposure to online risks. Recent internalizing symptoms were assessed using two *mental health indicators*: 1) depressive mood in the past 12 months (yes/no) and 2) frequency of self-harm thoughts (“thinking about you’d better off dead or of hurting yourself in some way”) in the past two weeks (never to nearly every day). Recent externalizing behaviors were assessed by using nine items asking *engagement in risk behaviors*, such as delinquency, risky sexual behavior, and substance use, in the past six months (yes/no). *Online risk exposure* was assessed using three items asking experiences of online teasing or cyberbullying, pornography consumption, and sexting (yes/no) (Supplementary Material). At the interpersonal level, p*arental monitoring* was assessed using an eight-item scale (22). Students rated each item on a five-point Likert scale indicating how often they shared whereabouts and activities with parents (e.g., “I talk to my parents about things that happen in school”). Item scores were averaged to create a composite parental monitoring score, with higher scores indicating a higher level of monitoring. The Cronbach's alpha of the scale was 0.81 in this study. At the environmental level, two indicators were included. First, neighborhood risk exposure was assessed with a nine-item scale measuring how often students observe risk behaviors in their neighborhoods (e.g., “How often do you see students ducking schools?”). Each item was rated on a three-point Likert scale, and item scores were averaged to create a neighborhood risk score, with higher scores indicating greater risk. The Cronbach's alpha of the scale was 0.74 in this study. Second, urban-rural residence was classified based on school location. Schools in New Providence (the capital island) were categorized as urban, as New Providence is the most urbanized, populous island. Schools on all other islands were categorized as rural.

### Analysis

A total of 2,357 students (approximately 95% of eligible Grade 9 students) completed the questionnaire. Descriptive statistics, chi-square tests, Cochran-Mantel-Haenszel tests, and Student's t-tests were used to detect significant differences in suicidal ideation. Spearman correlation was used to identify highly correlated variables within and across individual, interpersonal, and environmental levels. The Least Absolute Shrinkage and Selection Operator (LASSO) logistic regression was then used to select a parsimonious set of predictors and reduce multicollinearity. The final LASSO model was selected using the Schwarz Bayesian Criterion (SBC/BIC) in SAS PROC HPGENSELECT. Generalized linear mixed models with a binomial distribution and logit link function were subsequently used to assess the association between the selected variables and suicidal ideation adjusting for potential classroom- and school-level clustering effects. Classroom- and school-level random effects were evaluated but were omitted from the final model as they were not significant. Self-harm thoughts were removed from the model because of conceptual overlap with the primary outcome of suicidal ideation. The self-harm thought item captured thoughts related to both non-suicidal and suicidal self-injury and was strongly correlated with suicidal ideation (*ρ*=0.62). SAS 9.4 was used for data analysis.

## Results

Among the 2,357 students, 46% reported having ever seriously thought about killing themselves. Among those with lifetime suicidal ideation, 33% reported near-daily self-harm thoughts in the past two weeks. The rate of suicidal ideation among girls was approximately twice that of boys. All mental health indicators, risk engagement, online risk exposure, parental monitoring, and neighborhood risk were significantly associated with suicidal ideation in bivariate analyses, whereas urban-rural residence was not associated with suicidal ideation (Table 1).

**Table 1:** Bivariate associations between multilevel factors and lifetime suicidal ideation among Bahamian adolescents.

Variable	Reported SI (*N* = 1,104)	Not reported SI (*N* = 1,253)	*χ*^2^/*t*	p
	N (Column %) or Mean (SD)			
Total	46.8%	53.2%		
Gender				
Boys	370 (33.6%)	790 (63.2%)	205.6	<0.001
Girls	732 (66.4%)	460 (36.8%)		
Risk behavior (yes)^a^				
Delinquency				
Was truant	434 (39.6%)	370 (29.8%)	25.1	<0.001
Carried weapon	188 (17.1%)	150 (12.1%)	11.9	<0.001
Engaged in a fight	469 (42.8%)	429 (34.7%)	16.1	<0.001
Involved in robbery	96 (8.8%)	63 (5.1%)	12.5	0.001
Risky sexual behavior				
Had unprotected sex	155 (14.2%)	104 (8.4%)	19.9	<0.001
Touched someone inappropriately	340 (31.2%)	280 (22.8%)	20.8	<0.001
Substance use				
Smoked a cigarette	167 (15.2%)	115 (9.3%)	19.3	<0.001
Used marijuana	201 (18.4%)	120 (9.7%)	36.6	<0.001
Drank alcohol	671 (61.2%)	495 (40.0%)	104.4	<0.001
Online risk behavior				
Experienced cyberbullying	634 (58.4%)	442 (36.2%)	113.3	<0.001
Viewed or shared pornographic content	717 (66.4%)	568 (46.9%)	88.4	<0.001
Engaged in sexting	500 (47.0%)	350 (29.2%)	76.0	<0.001
Depressive moods (yes)^a^	913 (83.1%)	485 (40.4%)	439.6	<0.001
Parental monitoring^b^	3.9 (0.8)	4.0 (0.8)	−2.8	0.006
Neighborhood risks^c^	1.8 (0.3)	2.0 (0.4)	−8.3	<0.001
Region				
New Providence	749 (67.8%)	861 (68.7%)	0.2	0.65
Family Islands	355 (32.2%)	392 (31.3%)		
Self-harm thoughts in the past two weeks				
Nearly everyday	360 (32.9%)	41 (3.4%)	921.2	<0.001
Several days	241 (22.1%)	76 (6.3%)		
More than half of the days	327 (29.9%)	160 (13.3%)		
Never	165 (15.1%)	922 (76.9%)		

The mean age was 14.04 years and did not differ between students with and without suicidal ideation. Percentages were calculated using non-missing observations. SI, suicidal ideation.

a Frequences among students who reported “Yes” to each variable.

b Possible score range: 0–8.

c Possible score range: 0–9.

Multivariable analysis revealed that female gender, recent engagement in risk behaviors, exposure to online risks, and depressive moods were associated with a greater odds of reporting lifetime suicidal ideation (Table 2). Among individual-level factors, depressive mood, female gender, and experience of online teasing or cyberbullying had the strongest associations with suicidal ideation. Greater parental monitoring was associated with lower odds of reporting suicidal ideation (aOR 0.76, 95% CI 0.66-0.88), while neighborhood risk was not significant.

**Table 2:** Generalized linear mixed model assessing associations between multilevel factors and lifetime suicidal ideation among Bahamian adolescents.

Variable	Lifetime Suicidal Ideation		
	aOR	95% CI (LL, UL)	p
Age	0.98	(0.88, 1.10)	0.72
Gender			
Boys	1.00		
Girls	2.80	(2.24, 3.51)	<0.001
Risk Behavior			
No risk behavior	1.00		
Carried a weapon	1.44	(1.05, 1.99)	0.03
Engaged in a fight	1.24	(0.99 1.56)	0.06
Had unprotected sex	1.20	(0.85, 1.69)	0.29
Drank alcohol	1.26	(1.01, 1.56)	0.04
Experienced cyberbullying	1.97	(1.35, 2.88)	<0.001
Viewed or shared pornographic content	0.93	(0.63, 1.36)	0.69
Engaged in sexting	1.52	(1.21, 1.90)	<0.001
Depressive mood			
No depressive mood	1.00		
Depressive mood	5.00	(4.01, 6.22)	<0.001
Parental monitoring	0.76	(0.66, 0.88)	<0.001
Neighborhood risk	0.88	(0.64, 1.20)	0.41

aOR, adjusted odds ratios; CI, confidence interval; UL, upper limit; LL, lower limit.

## Discussion

This study found an alarmingly high prevalence of suicidal ideation among Bahamian adolescents in public schools (46%). The rate far exceeded the prevalence of lifetime suicidal ideation among children and adolescents in Africa (37%), which was highest reported among all continents in a systematic review (23). Data collection occurred during the COVID-19 pandemic recovery period, which may have contributed to the elevated rate, but the prevalence in this study was still more than twice the global adolescent average of 22% reported during the pandemic (24). This elevated rate may reflect contextual and social stressors affecting Bahamian adolescents during this period. Given that adolescent suicidal ideation significantly increases the risk of suicide attempts in young adulthood (25), and that depressive symptoms were strongly associated with suicidal ideation, these findings underscore the importance of strengthening mental health support among Bahamian adolescents.

Among individual-level factors other than depressive mood, online teasing or cyberbullying had the strongest association with adolescent suicidal ideation. As social media plays an increasingly central role in adolescents’ lives, their exposure to online risk is increasing. Online risk exposure may have a greater impact on mental health than offline risk behavior engagement, as anonymity, invisibility, and lack of immediate consequences can intensify aggressive behaviors in digital settings (26, 27). A recent meta-analysis found cyberbullying to have a stronger effect on self-harm and suicidal ideation than offline bullying (28), highlighting that online risks, often less visible to adults, play an increasingly important role in adolescents’ mental health. Interventions should prioritize online behaviors as a critical and modifiable risk factor, and adolescent mental health programs should include regular monitoring and targeted support for affected adolescents.

Beyond the individual level, our study highlights the importance of interpersonal influences: we found that parental monitoring was protective against suicidal ideation, consistent with previous research (11). The increase in social media use and online risk exposure underscores the need for parental monitoring of adolescents’ online activities. However, effective strategies for such parental involvement remain underexplored. One study found that restricting internet access decreased exposure to online abuse (29), whereas another study reported that parental monitoring of internet use alone did not mitigate its negative effects on adolescents’ mental health (30). Further research is needed to identify effective forms of parental supervision of online behavior.

In contrast, we did not find a significant independent association between environmental factors and adolescent suicidal ideation. Although neighborhood risk was significant in bivariate analyses, it was no longer significant after adjusting for other variables, including risk behaviors. This suggests that adolescents’ engagement in risk behaviors may play a more critical role than neighborhood context alone.

This study has several limitations. Temporal ambiguity arises from the use of different recall periods across measures: Suicidal ideation was measured as a lifetime event to broadly identify adolescents at risks, while other psychopathological and behavioral symptoms were assessed using shorter recall periods to reduce recall bias. Further, a single item measured suicidal ideation, limiting evaluation of its frequency or severity. The cross-sectional design limits findings to associations rather than causal risk pathways. Because lifetime suicidal ideation was relatively common in this sample, odds ratios may overestimate true prevalence ratios. Unmeasured school environment factors, such as school connectedness or peer support, may also influence suicidal ideation. Finally, findings may not generalize to private school students or other age groups.

In conclusion, our findings underscore the need for tailored, multilevel strategies that address both online and offline influences on adolescent mental health. Given that parental monitoring was a protective factor for suicidal ideation, multilevel interventions that involve both students and their parents may be more effective than those targeting adolescents alone. School-based programs that engage students and parents in promoting healthy online behavior, preventing cyber risks, fostering school-parent collaboration in supervision and monitoring, and providing mental health support may offer a promising approach to reducing suicidal ideation (31).

## Data Availability

The raw data supporting the conclusions of this article will be made available by the authors, without undue reservation.
