# Supplementary material for: High prevalence and multilevel correlates of suicidal ideation Among Bahamian adolescents: findings from a national study
Source: Front Child Adolesc Psychiatry. 2026 Jul 16;5:1848813. doi: 10.3389/frcha.2026.1848813 (PMC13422466; doi:10.3389/frcha.2026.1848813)
Supplement: Supplementary file 1 [file Datasheet1.pdf]

Supplement 1. Measures

Psychopathological Symptoms

Depressive mood

During the past 12 months, did you ever feel so sad or hopeless almost every day for two weeks or more in a row that you stopped doing some usual activities? ☐ Yes ☐ No

Self-harm thoughts

How often in the past two weeks have you had thoughts that you would be better off dead or of hurting yourself in some way?

☐ Never ☐ Several days ☐ More than half of the days ☐ Nearly every day

Risk behavior

	Yes	No
Delinquency		
1. In the last six months there were times I missed school because I did not feel like going (I ducked school).		
2. In the last six months, I did carry a knife, screwdriver or cutlass to use as a weapon.		
3. In the last six months, I did fight with someone, other than with my brother or sister.		
4. In the last six months, I was involved in stealing or breaking into a home, shop or business.		
Risky sexual behavior		
5. In the last six months, I put myself in a position where I could have become pregnant, make someone pregnant or become infected with HIV.		
6. In the last six months, I (did feel up) or touch someone in a wrong way.		
Substance use		
7. In the last six months, I have smoked a cigarette or Backwoods.		
8. In the last six months, I have used marijuana (weed).		
9. In the last six months, I did drink beer, rum, liquor or wine (not including wine taken during communion at church).		

Online risk behavior

	Yes	No
1. Have you ever experienced teasing or cyberbullying?		
2. Have you ever viewed pornographic (sexually explicit) pictures or videos on the internet?		
3. Have you ever taken and/or shared a pornographic picture or video over the internet?		
4. Have you ever engaged in sexting (sexually explicit text messaging)?		

Parental Monitoring

	Never	Hardly Ever	Sometimes	Most of the Times	Always
1. I talk to my parents about things that happen in school.					
2. If I'm going to be home late, I tell my parent(s) or guardian.					
3. Before I go out, I tell my parents who I am going to be with.					
4. If I am out at night, this means that I am out with my parents or guardians or someone they know and trust.					
5. I talk to my parents or guardians before I make plans to do something with my friends.					
6. When I go out, I tell my parents or guardians the truth about where I am going.					
7. When I go out, I come back home at the time my parents/guardians say that I should.					
8. When I get home from school, I let my parents or guardians know that I am home.					

Environmental Factors

Neighborhood risks

	Very often	Sometimes	Never
1. How often do you see students ducking schools?			
2. How often do you see someone carrying a knife, screwdriver or cutlass to use as a weapon?			
3. How often do you see someone carrying and/or using a gun?			
4. How often do you see someone breaking into a car, home, shop or business?			
5. How often do you hear of a teenager becoming pregnant or getting someone pregnant?			
6. How often do you see people drinking beer, rum, liquor or wine at any time (not including wine taken during communion at church)?			
7. How often do you see people using or selling marijuana(weed)?			
8. How often do you see young people being touched inappropriately (getting felt up) without their permission?			
9. How often do you see people fighting?			
